# Engineering Human Donor Derived Germinal Center‐Like Organoids (GCLOs) for Studying Immune Response to Vaccination

**DOI:** 10.1002/advs.76335

**Published:** 2026-07-03

**Authors:** Bhumi Suthar, Carlos Gomez, Giancarlo Asencio, Suresh Pallikkuth, Ashutosh Agarwal

**Affiliations:** ^1^ Department of Biomedical Engineering University of Miami Coral Gables Florida USA; ^2^ Microbiology and Immunology University of Miami Miller School of Medicine Miami Florida USA; ^3^ Desai Sethi Urology Institute University of Miami Miller School of Medicine Miami Florida USA

**Keywords:** b cell, biology, cd19, germinal center, humoral immunity, immune system, immunology, immunosenescence, peripheral blood mononuclear cell

## Abstract

Germinal centers (GCs) are transient microanatomical structures that coordinate humoral immune responses through dynamic interactions between antigen presenting cells, T follicular helper cells, and B cells. Despite their central importance to immune protection, GC human biology remains difficult to interrogate due to significant inter‐species variability, limited experimental accessibility to primary human tissues, and high interindividual variability. We developed a human immune culture platform that supports spontaneous multicellular organization of human peripheral blood mononuclear cells derived from primary CD19+ B cells, memory CD4+ T cells, and monocyte‐derived dendritic cells into Germinal Center‐like Organoids (GCLOs). These bioengineered GCLOs self‐organize into stable clusters that exhibit stimulus and donor dependent differences in cellular composition and function. We observed the emergence of GC‐like T and B cell subsets, functional IgG output, and the appearance of plasmablast‐like populations. Notably, the age of the donors and the type of stimuli influenced GCLOs architecture, immune subsets, and antibody output. Platform outputs showed correspondence with clinical vaccine responsiveness across human donors, capturing inter‐individual differences in humoral GCLO immune responses to the flu vaccine, including age‐associated trends consistent with immunosenescence. This platform allows scalable investigation of human GC‐associated immune features and provides a tractable framework for studying donor heterogeneity in humoral immunity.

## Graphical abstract

We bioengineered a new approach methodology of generating Germinal Center Organoids that self‐organize from human blood‐derived immune cells. These immune organoids reproduce key features of humoral immunity, IgG production, and plasmablast emergence. Donor age and vaccine stimuli shape organoid architecture and vaccine responsiveness, revealing interindividual variability and immunosenescence‐associated trends. The platform enables scalable studies of germinal center biology.

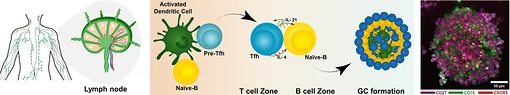

## 1 Introduction

Protective humoral immunity depends on the formation of germinal centers (GCs); highly organized and dynamic structures that arise within secondary lymphoid organs following infection or immunization. Following antigen exposure, dynamic interactions between dendritic cells, CD4+T follicular helper (Tfh) cells, and B cells drive clonal selection, class switch recombination, somatic hypermutation, affinity maturation, and differentiation into memory B and plasma cells to produce high‐affinity antibodies (Figure 1A) [1, 2, 3]. Neonates and young infants exhibit immature GCs with limited somatic hypermutation and Tfh support, often leading to weaker humoral responses. In contrast, older adults may exhibit altered GC function due to immunosenescence, characterized by reduced Tfh support, diminished B cell proliferation, and impaired class‐switch recombination, contributing to lower vaccine efficacy.

**FIGURE 1:**
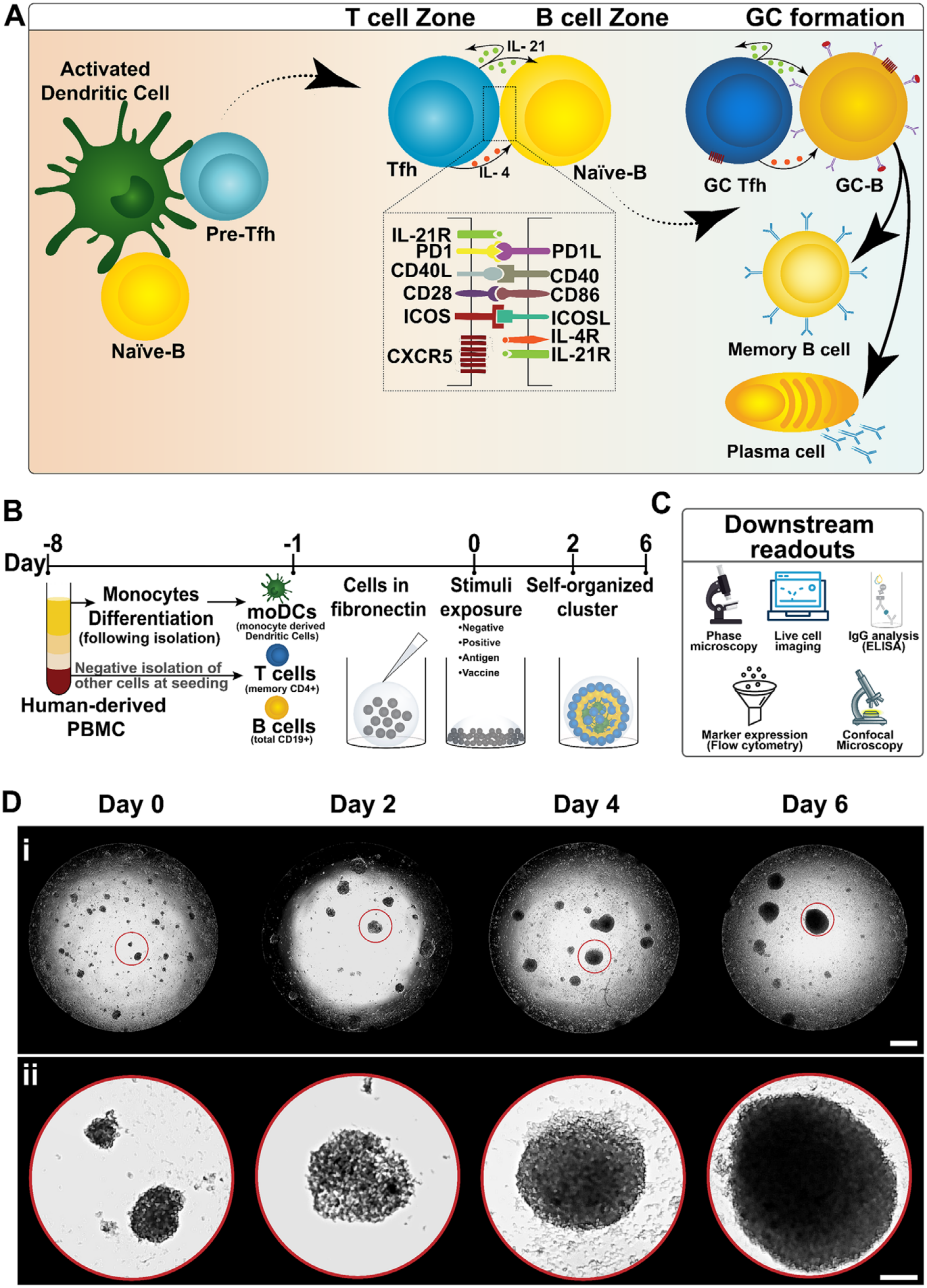
Germinal center biology and design principles of the human‐based immune platform. (A) Schematic illustration of key cellular interactions underlying a GC response. Dendritic cells present antigen to naïve B cells and pre‐Tfh CD4+ T cells, leading to Tfh differentiation and B cell activation. Interactions between Tfh cells and naïve B cells occur at the T–B border, followed by migration of T&B intermediate subsets to the GC forming region. Receptor‐ligand interactions central to Tfh‐B cell communication are highlighted in the inset. Within the GC‐like niches, Tfh cells interact with antigen‐engaged B cells, supporting B cell activation and differentiation. The fate decision toward memory B cells and antibody‐secreting plasma cells is determined by the GC T & B subsets. Within the schematic, dotted arrows represent cell migration, and solid arrows represent cell differentiation. (B) Representation of the experimental workflow. Monocytes were isolated from primary human PBMCs (peripheral blood mononuclear cells) on day ‐8, followed by differentiation into dendritic cells (monocyte‐derived dendritic cells (moDCs)). On day ‐1, the moDCs are harvested, and memory CD4+ T cells & CD19+ B cells are isolated. The three cell types were suspended in a final concentration of 1 mg/mL fibronectin (bovine‐derived) suspension at a 1:1:1 ratio and co‐cultured in a 96‐well format on day ‐1. On day 0, the platform is exposed to no stimuli or Negative (media only) or defined immune stimuli as follows: Positive (5 µg/mL anti‐CD40 + 1 µg/mL IL‐4), Antigen (1 µg/mL SEB (staphylococcal enterotoxin B), and Vaccine (2.64 µg/mL Fluzone). We observed initiation of GCLO formation from day 2 upon stimulation, which expanded further until day 6, depending on the type of stimulus and donor. Media supernatant was collected every alternate day for downstream IgG analysis and replenished with fresh complete RPMI‐1640 medium. (C) Summarized downstream phenotypic and functional readouts. Phase contrast and live cell imaging were conducted to monitor the progress of GCLO formation, expansion, and immune interactions. The media supernatants were used for analyzing the IgG production in various conditions. On day 6, the cells were harvested from the platform, and phenotypic markers were quantified using flow cytometry. All conditions were performed in triplicate (3 wells per condition in the 96‐well format), and one of each condition was used in confocal laser scanning microscopy to evaluate organization architecture of GCLOs. (D) Representative phase‐contrast images display the emergence, growth, and self‐assembly of the GCLOs over time. (i) Scale bar represents 1 mm. (ii) Scale bar represents 200 µm.

By incorporating relevant cell types such as T cells, B cells, dendritic cells, and macrophages, and recreating their complex cellular interactions and microanatomical organization, engineered in vitro GC models can simulate the lymph node microenvironment. Ranging from lymph‐node‐on‐chip systems to tonsil organoids, they can more accurately allow GC formation, B/T cell dynamics, plasma cell differentiation, and antibody production than traditional culture systems. Antibody responses depend upon optimal generation of antibody‐secreting B cells through critical interactions with antigen primed T follicular helper cells within germinal centers of peripheral lymphoid tissues [4, 5, 6, 7, 8, 9, 10, 11]. This intricate cellular choreography relies on the extracellular matrix (ECM), which creates conduits that transport signaling molecules while maintaining compartmentalized immune niches [12]. Despite extensive in vivo evidence linking ECM organization to GC biology, most in vitro immune models either lack defined ECM components or rely on complex stromal co‐cultures and engineered architectures that limit scalability and mechanistic interpretability [13, 14, 15]. Other studies either use a composite of ECM proteins (such as Matrigel, whose composition varies based on donor and extraction efficiency) [4, 5, 14, 16, 17, 18, 19]. The heterogeneity arising with such microenvironments cannot be attributed solely as immunological. Thus, we examined a minimal ECM‐based platform composed of fibronectin to support immune cell coordination in vitro, in the absence of predefined tissue architecture or stromal networks.

After testing out different ECM formulations, we utilized a minimal ECM composed solely of fibronectin to probe spontaneous immune self‐organization and functional humoral responses in vitro without predefined tissue architecture. Fibronectin, a major ECM glycoprotein, is highly abundant during immune activation and inflammation and serves as a mechano‐responsive scaffold that supports leukocyte adhesion and migration through integrin‐mediated interactions [12, 20]. The platform incorporates primary human monocyte‐derived dendritic cells, memory CD4+ T cells, and CD19+ B cells suspended in fibronectin matrix and placed in a standard multiwell plate. We observe self‐organized cluster formation that mimics GC‐like biology and functional immune output upon stimulation with defined immunological cues. Across stimulation conditions, we observed distinct and reproducible immune cell organization and population. The distinct cellular and functional phenotypes across stimulation conditions highlight the platform's sensitivity to physiological cues. The study uses cells derived from six healthy donors, including three older adults (A1, A2, A3) and three younger adults (Y1, Y2, Y3), all of whom received a standard dose seasonal influenza vaccine. We used the Fluzone lots corresponding to the same influenza season in which each donor was vaccinated. Donor's clinical titers and responsiveness to the vaccine was mirrored by the platform's IgG concentration. Vaccine stimulation elicited the most inter‐donor variability, underscoring the importance of examining immune responses at the individual level. The IgG accumulation, along with UMAP‐derived cellular compositions, illustrates a tight correspondence between platform and clinical outcomes.

Overall, the platform supports cognate B cell – T cell interactions, plasmablast differentiation, and antibody secretion over a clinically relevant time frame. Human GCLOs enable a framework for studying donor‐specific secondary humoral responses, probing age‐associated immune decline, capturing and predicting human‐specific vaccine responses, and immune heterogeneity.

## 2 Results

### 2.1 GCLO Self‐Organization and Expansion

This study included deidentified PBMC samples from six healthy adult donors, who received seasonal influenza vaccination, comprising three older (A1, A2, A3) and three younger (Y1, Y2, Y3) individuals. Each donor's influenza season and vaccine response titers were documented. For the vaccine condition, Fluzone lots corresponding to the same influenza season as each donor's vaccination were used. Primary monocytes were isolated from PBMC and differentiated to yield monocyte‐derived dendritic cells (moDCs), which were then co‐cultured with memory CD4+ T cells and total CD19+ B cells in fibronectin suspension. The design was intended to determine whether fibronectin alone is sufficient to permit immune‐driven self‐organization. Our optimization experiments utilized immune cells such as Monocytes (CD11c+/CD14+), T cells (CD3+), & B cells (CD19+) positively isolated from human donor‐derived PBMCs (peripheral blood mononuclear cells) with flow cytometry (Figure S1A). Monocytes played the role of APCs (antigen presenting cells). Isolated immune cells were co‐cultured on a 48‐well plate in their respective microenvironments. We tested Lyophilized Gelatin Scaffold (LGS), which was 5 mm in diameter and 200 µm thickness and ∼50 µm pore size, Collagen solution‐ 3.7 mg/mL of rat tail collagen IV was used, Fibronectin: collagen solution: 1:1 ratio, Fibronectin solution: 1 mg/mL of bovine‐derived fibronectin was used. Apart from the collagen matrix, GCLO‐like clusters were observed in all microenvironments when stimulated with 5 µg/mL anti‐CD40 + 1 µg/mL IL‐4 (Figure S1B). IgG concentration accumulated in the culture supernatant of various microenvironments was quantified using ELISA. Although each matrix produced detectable IgG, a sustained IgG concentration was observed in immune cells suspended in fibronectin. (Figure S1C).

We observed spontaneous emergence of multicellular clusters that increased in size and density over the culture period (Figure 1D). Phase contrast imaging revealed progressive cellular assembly, resulting in compact three‐dimensional GCLO structures. GCLO formation was donor‐specific and reproducible across stimulus, indicating that organization was not driven by stochastic aggregation but instead reflected coordinated cellular behavior within the fibronectin matrix. Notably, cluster expansion occurred without the inclusion of stromal cells, follicular dendritic cells, or exogenously imposed chemokine gradients. This suggests that cell‐intrinsic migratory programs, along with ECM‐mediated interactions, support immune cell organization in this system. Consistent with the role of fibronectin [20, 21, 22, 23], our system demonstrated its function as a supportive and instructive scaffold that enables immune cells to explore, interact, and self‐assemble.

The observations establish that a single‐component ECM can support immune cell organization in vitro. The platform thus presents a simplified yet biologically relevant foundation to probe immune cell interactions, phenotypic transitions, and functional outputs emerging from collective behavior, which we examine further.

### 2.2 Donor‐Resolved and Stimulus Dependent Cellular Composition Emerges Within GCLOs

To assess whether GCLOs displayed features associated with germinal center (GC) biology, we examined cellular organization across conditions. Analyses were performed at day 6 of the experiment, corresponding to a time point at which GCLOs were structurally stable and functionally active. Phase contrast imaging (Figure 2A.i) suggested dense, well‐defined GCLOs, while confocal laser scanning microscopy (Figure 2A.ii) unveiled cellular proximity and spatial architecture of B (CD19 green) and T (CD3 red) lymphocytes within individual GCLOs. We observed B and T cells occupying defined spatial domains rather than forming random or homogeneous aggregates. Additionally, the architecture of the GCLOs varied in a stimulus‐dependent manner within the platform. The anti‐CD40 + IL‐4 condition models T cell–dependent B‐cell activation by providing CD40–CD40L–like stimulation and cytokine cues that support B‐cell survival, class‐switch recombination, and differentiation. In the absence of antigen and dendritic cells, B cells under this condition display a more diffuse distribution within the structure. In contrast, under antigen (SEB) conditions, consistent with the superantigenic properties of SEB, enhanced T–B cell interactions are observed, resulting in increased Tfh cell proliferation. In the vaccine condition, optimal T–B–APC interactions are evident, with the emergence of a more organized architecture characterized by a distinct B‐cell zone surrounded by T cells and APCs, consistent with a GC–like organization. These observations suggest that the platform supports not only cluster persistence but also sustained immune cell colocalization at later time points compatible with GC‐like cell‐to‐cell communication. Binary grayscale representations of the same GCLO further highlighted the degree of intermixing and spatial overlap between immune subsets, suggesting active communication and coordination within the microenvironment. (Figure S2). Dendritic cells (CD14 blue) were mostly observed in the periphery of the GCLOs. The density and involvement of DCs varied across stimuli (Figure S3).

**FIGURE 2:**
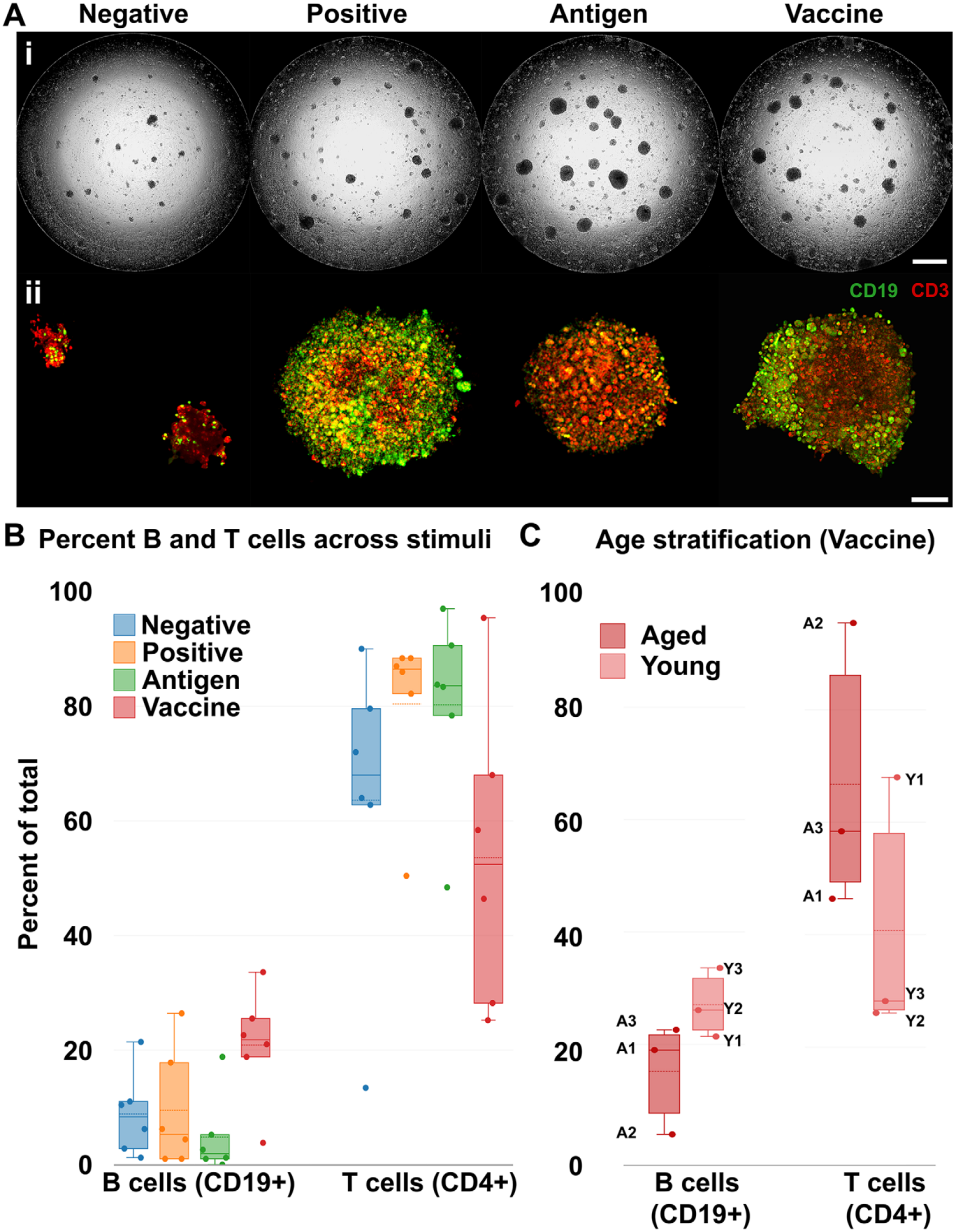
Fibronectin Microenvironment‐supported GCLOs Exhibit Donor and Stimulus‐Specific Cellular Composition. (A) Phase contrast (i) and confocal (ii) microscopy images of GCLOs on day 6 unveil the varied spatial composition of B & T cells across conditions. GCLOs were fixed, permeabilized, and stained with CD19 & CD3 as a marker for B & T cells, respectively. (i) Scale represents 1 mm. (ii) Scale bar represents 100 µm. (B) Quantification of relative proportions of B & T cells across donors and stimuli using flow cytometry. The percentage of CD19+ (B cells) & CD4+ (T cells) were measured using flow cytometry across stimuli. Positive and antigen conditions drive progressively greater T cell dominance, whereas vaccine stimulation preserves a relatively greater B cell proportion. Each point represents an individual donor (*n* = 6; 3 young and 3 aged). (C) Age‐stratified analysis of the vaccine condition: A higher percentage of T cells compared to B cells in aged donors vs young donors was observed. Each point represents an individual donor (*n* = 6; 3 young and 3 aged). The dotted line within the box represents the mean, and the solid line represents the median. The upper and lower bars represent the minimum and maximum values, respectively. No formal statistical analysis was applied due to the low sample size.

To assess how different stimuli shape cellular organization within GCLOs, we examined B and T cells representation across conditions that differ in their modes of T cell activation (Figure 2B). The relative abundance of CD19+ B and CD4+ T cells across stimuli and donors is quantified using flow cytometric analysis on day 6 of the experiment. Since DCs serve primarily as early instructional cells and were not a dominant component of mature GCLOs, downstream flow cytometry analyses focused on B and T cell populations. The data is presented in a donor‐specific manner to illustrate inter‐individual variability rather than population‐level differences. (i) Negative condition (no stimulation) exhibited a T cell dominant composition with comparatively fewer B cells, indicating that basal self‐organization within fibronectin favors T cell persistence. In the absence of exogenous cues, B cells receive insufficient survival or activation signals to sustain their persistence in the culture, (ii) Positive condition (5 µg/mL anti‐CD40 + 1 µg/mL IL‐4) provides both co‐stimulatory (CD40) and cytokine (IL‐4) signals that broadly support T cell activation and B cell differentiation. We observed a more pronounced skew towards T cells, consistent with strong co‐stimulatory signals (CD40 engagement) that preferentially activate and enhances survival of T cells, (iii) Antigen condition (1 µg/mL Staphylococcal enterotoxin B (SEB)) is a bacterial superantigen that engages T cell receptors and co‐stimulatory pathways to induce polyclonal T cell activation, leading to a marked expansion of T cells relative to B cells [24, 25, 26]. We observed a skewed composition toward T cell prevalence and lower B cell participation within our platform, (iv) Vaccine (Fluzone) condition produced a distinct cellular proportion in which B cell representation was preserved relative to both positive and antigen conditions. This vaccine‐specific profile suggests a more coordinated B–T engagement, consistent with helper T cell support for B cell retention and potential differentiation signals.

Given the age‐associated changes in human humoral immunity [17, 27], we next explored whether donor age influenced GCLO composition in response to stimuli. Age stratification was centered on the vaccine condition as it represents a clinically standardized immune challenge with well‐documented age‐associated trends (Figure 2C). Although the sample size is limited, GCLOs derived from young and aged donors exhibited distinct distribution patterns in B and T cell representation, suggesting age‐associated trends. In young donors, B and T cells consistently comprised typically 25%–40%, reflecting a relatively balanced proportion of the two‐lymphocyte population. In contrast, aged donors exhibited a shift toward higher T cell (∼50%) and lower B cell proportion (<20%) (Figure 2C). Despite this compositional shift, organized B‐ and T‐containing clusters were observed across all donors (including non‐responders). Notably, one aged donor (A1) displayed immune composition comparable to the young donor range, consistent with the antibody responsiveness of the individual to the vaccine (Figure 2C). While these observations are descriptive in nature and not intended as statistically powered comparisons, they qualitatively align with reported features of age‐associated immune remodeling and immunosenescence. Overall, the data suggest that GCLOs formed with minimal ECM environment remain compositionally dynamic and responsive to both external stimuli and donor‐specific immune variability. This donor‐ and stimulus‐specific organization provides a foundation for subsequent analysis of GC‐associated phenotypes and functional immune output.

### 2.3 Characterization of Follicular‐Associated Phenotypic Markers and Cell States

We evaluated whether the platform supported differentiation into phenotypic features associated with GC reactions. Markers CXCR5 and CD27 were selected to capture complementary aspects of follicular organization and B cell differentiation. CXCR5 directs T and B cell migration toward CXCL‐13‐rich niches and supports sustained T‐B interactions characteristic of germinal center environments. CD27 marks antigen‐experienced Tfh cells with superior helper function, while CD27 expression on B cells is associated with memory and plasmablast differentiation trajectories. Combined analysis of these markers enables assessment of follicular‐associated organization within GCLOs without presupposing germinal center identity. The expression of key follicular‐associated markers on T and B cells was analyzed on day 6, with marker frequencies expressed as a percentage of total T or B cells, respectively. CXCR5+ B cells were most enriched under positive conditions (∼60%), while antigen and vaccine stimulation in comparable intermediate frequencies (∼40%), and unstimulated cultures showed slightly higher expression (∼45%) (Figure S4A). In contrast, CD27+B cells were most abundant under negative conditions (>80%), remained high under positive and vaccine conditions (∼70%), and were markedly reduced under antigen stimulation (∼30%–40%) (Figure S4A). This is in contrast to the cluster composition data, which indicates a higher abundance of B cells in the positive antigen, and vaccine conditions compared with the negative condition. It may be possible that pre‐existing memory B cells preferentially survive in culture even without antigen stimulation, whereas naïve and other B cell subsets may have reduced survival due to the absence of T cell help. As a result, the relative frequency of memory B cells would appear increased because they represent the surviving population. Notably, in stimulated conditions, many B cells spatially overlap with Tfh cells, consistent with active T–B interactions. In contrast, such interactions are largely absent in the negative condition, where B cells appear more isolated. This pattern suggests that follicular‐homing cues and T–B contact may promote B‐cell retention, survival, or phenotypic maturation in the stimulated settings. The CD27^+^ B cells observed under the negative condition may therefore reflect either pre‐existing memory B cells present at baseline or spontaneous acquisition of activation markers in the presence of T cells, rather than de novo GC–driven differentiation. Overall, these data highlight the importance of productive T–B interactions in shaping B‐cell fate within the system.

We quantified CXCR5 expression on CD4+ T cells. They were least frequent under unstimulated conditions (<20%), increased substantially under antigen stimulation (∼40%), and were present at comparable levels under positive and vaccine conditions (∼25%–30%) (Figure S4A). These findings indicate that T cell follicular positioning cues are stimulus‐sensitive within the platform. The antigen exposure driving the strongest CXCR5 induction, while vaccine and positive control conditions promote intermediate T cell follicular‐homing phenotypes. This divergence suggests that distinct stimuli preferentially bias B cell follicular positioning versus differentiation states within the same microenvironment.

Multiplex immunofluorescence imaging demonstrates spatial co‐localization of CXCR5 expressing T and B cells alongside CD27+ and CD16+ cells (Supplementary Figure S4B). These spatial arrangements are consistent with follicular‐like microenvironments that support coordinated T–B interactions. Age‐stratified analysis under the vaccine condition revealed selective enrichment of CXCR5+ T cells in aged donors, whereas CXCR5+ B cells and CD27+ B cells were more abundant in young donors (Figure S4B). A spatial profile of the markers can be observed in the confocal image in Figure S4C. This pattern is consistent with age‐associated alterations in B–T coupling, in which follicular positioning cues in T cells may be preserved or enhanced, while follicular‐associated B cell states are comparatively diminished. The ability of the platform to reveal such divergent age‐dependent phenotypic biases highlights its sensitivity to donor‐specific immune differences.

### 2.4 Functional Antibody Production by GCLOs

To determine whether the immune organization and follicular‐associated phenotypes translate into functional humoral output, we quantified secreted IgG levels across stimulation and donors. Supernatants were collected on days 2, 4, and 6 to analyze the IgG secretion, and the concentrations were measured using ELISA. The absolute IgG concentrations (ng/mL) are shown as heatmaps faceted by conditions (Negative, Positive, Antigen, Vaccine) to capture dynamic changes over time while preserving donor‐specific (*n* = 6) kinetics. Donor labels A1–A3 and Y1–Y3 designate aged donors 1–3 and young donors 1–3, respectively (Figure 3A). Given the small sample size and single‐well measurements per condition, no statistical comparisons were made. The analysis thus focuses on descriptive trends and donor‐specific trajectories rather than definitive.

**FIGURE 3:**
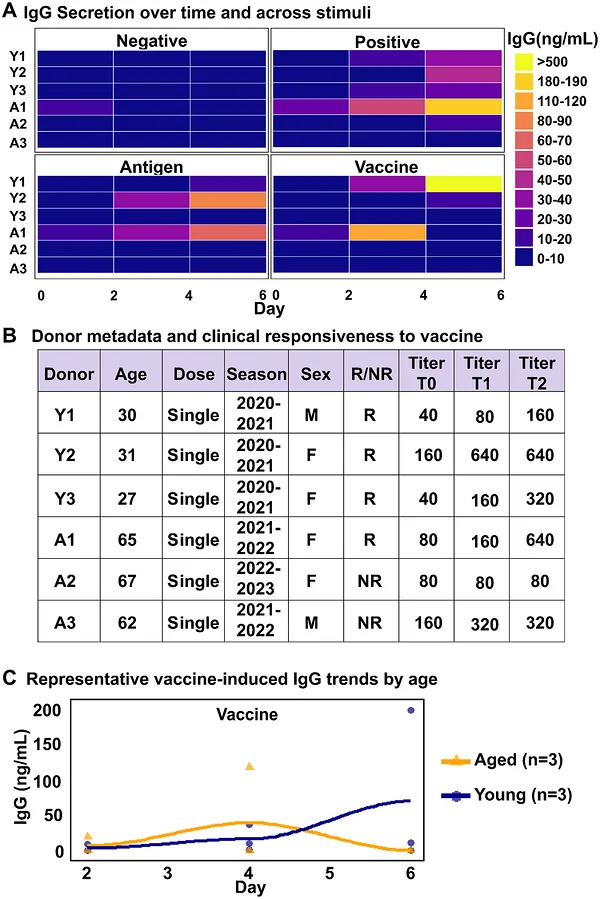
Donor‐specific variability in IgG production across stimuli and correlation with clinical vaccine responsiveness. (A) Heatmap of IgG concentration (ng/mL) for individual donors and distinct stimulus over time. Donor labels A1–A3 and Y1–Y3 designate aged donors 1–3 and young donors 1–3, respectively. Negative (no stimuli) condition observed a steady and minimal to no IgG over time, indicating the absence of spontaneous differentiation. In contrast, the positive (5 µg/mL anti‐CD40 + 1 µg/mL IL‐4) condition observed a progressive increase in IgG over time across most donors, with the highest concentration being ∼180 ng/mL. Antigen (1 µg/mL SEB (staphylococcal enterotoxin B)) condition elicited a modest but gradual increase in IgG over time, with the highest concentration being ∼80 ng/mL, vaccine (2.64 µg/mL Fluzone) condition resulted in heterogeneous IgG trajectories, with some individuals showing clear increases whilst others exhibiting minimal responses, highlighting inter‐individual variability. (B) Donor information and clinical response to vaccine. The table summarizes, for each donor, age group, vaccine dose & year, sex, clinical response classification (responder (R), non‐responder (NR)), vaccine‐specific serum antibody titers at baseline (T0) and post vaccination time points (T1, T2). Donors who mounted a clear increase in vaccine‐specific serum titers in vivo generally exhibited strong vaccine‐induced IgG production in the platform, whereas clinical non‐responders showed minimal IgG induction. (C) Representative vaccine‐induced IgG trends by age. LOESS (Locally estimated scatterplot smoothing) regression trajectories of vaccine‐induced IgG secretion stratified by age group illustrate inter‐donor variability within and between age classes. Although the sample size is low to conclude age‐related differences, the platform does reveal response trajectories consistent with the known features of immunosenescence, with aged individuals tending toward reduced functional IgG output.

In negative condition, across donors, the IgG levels remained largely stable over time, indicating baseline antibody secretion in the absence of exogenous stimulation. Positive condition (5 µg/mL anti‐CD40 + 1 µg/mL IL‐4) induced a consistent progressive increase in IgG concentration across most donors, reflecting robust polyclonal B cell activation and differentiation. Antigen (1 µg/mL SEB, staphylococcal enterotoxin B) condition elicited a modest but gradual increase in IgG over time. The observation is consistent with SEB's role as a superantigen that induces a robust polyclonal T cell activation, which may be insufficient to support sustained B cell survival and maturation into antibody‐secreting cells [24, 25, 26]. The relative proportions of B and T cells as quantified in Figure 2B aligns with and support the IgG secretion patterns. The vaccine (2.64 µg/mL Fluzone) condition yielded heterogeneous IgG trajectories, with substantial variability in magnitude and kinetics among donors. While some individuals exhibited clear and progressive increases, others displayed minimal or delayed responses. The results highlight pronounced inter‐individual variability in functional vaccine responsiveness (Figure 3A).

We questioned whether the donor heterogeneity observed in vaccine response represented a biological phenomenon or variability introduced by the platform. To address this, we compared in vitro IgG secretion profiles with donor metadata and clinical antibody titers. The individuals were classified as responders(R) or non‐responders (NR) based on in vivo increases in vaccine‐specific serum titers between baseline (T0) and post‐vaccination time points (T2) (Figure 3B). Vaccine responders were defined as participants with a baseline HAI titer of 1:40 or lower who demonstrated a ≥ 4‐fold increase in post‐vaccination antibody titers [28]. Several clinically defined vaccine responders exhibited elevated vaccine‐induced IgG secretion, whereas clinically defined non‐responders generally exhibited limited IgG accumulation in the platform over a period of 7 days (Figure 3A,B). Notably, one clinical responder (Y3) represented a clear outlier, showing low IgG accumulation in the culture supernatant (Figure 3A). However, our flow cytometry analysis revealed the presence of a decent proportion of plasmablasts (Figure 5B), suggesting that while the GCLO platform successfully initiated B cell differentiation for this donor, the kinetics of antibody secretion may lag in vitro. This observation underscores that the platform captures donor‐specific mechanistic blocks that may be bypassed in vivo by systemic factors. The vaccine‐induced IgG trajectories were stratified by age using LOESS (Locally estimated scatterplot smoothing) regression. We observe diverse secretion patterns across donors, and aged donors overall display a lower IgG output compared to young donors. The trends are qualitatively consistent with the features of immunosenescence. Overall, these findings suggest that the platform may capture selective aspects of donor immune responsiveness rather than fully reproducing the magnitude or kinetics of clinical responses across all individuals.

### 2.5 Stimulus‐Dependent UMAP Visualization of Immune Cell Populations

To characterize the effect of various stimulations in shaping immune cell composition within the platform, high‐dimensional flow cytometry data from six donors were concatenated and visualized using UMAP. The immune subsets projected are T cells (CD4+), B cells (CD19+), Tfh‐like cells (CD4+ CXCR5+ IL21R+ ICOS+ PD1+), and Plasmablasts (CD19+ CD20+ CD27+ CD38+) (Figure 4). The gating strategy employed to identify each subset is mentioned in Figure S3. In a negative condition, the immune landscape is dominated by T cells, with detectable plasmablasts and sparse B cells. This profile reflects the baseline composition of isolated immune cells rather than active differentiation, consistent with a quiescent immune state in the absence of external stimulation. A small number of Tfh‐like cells were detected, suggesting the carryover of pre‐committed cells rather than de novo differentiation (Figure 4). The immune subsets align with our prior observation of low IgG detection. The positive condition (anti‐CD40 + IL‐4) resulted in a distinct remodeling of the immune landscape, characterized by an expanded T cell compartment with an increased proportion of Tfh‐like cells and a relative enrichment of B cells compared to the negative condition. The presence of helper‐like T cells, together with increased B cell abundance, provides a mechanistic basis for the robust and progressive IgG secretion observed. These changes are consistent with the provision of strong co‐stimulatory (CD40 ligation) and cytokine signals (IL‐4) that promote lymphocyte activation and differentiation. Antigen (SEB (staphylococcal enterotoxin B)) condition produced a landscape largely dominated by T cells, the majority of which displayed a Tfh‐like phenotype, and fewer B cells and plasmablasts. This pattern is consistent with the known activity of superantigens in inducing strong, polyclonal T cell activation that may not reliably sustain cognate B‐T interactions required for robust B cell maturation and differentiation. Vaccine (Fluzone) condition generated a qualitatively distinct immune landscape, with proportionate representation of B and T cells and the highest relative abundance of plasmablasts. This profile reflects physiological, antigen‐driven cognate interactions that coordinately activate and differentiate memory B cells into antibody‐secreting cells (Figure 4). Overall, the data demonstrated that the distinct stimulation conditions drive reproducible and biologically interpretable remodeling of immune cell composition within the platform.

**FIGURE 4:**
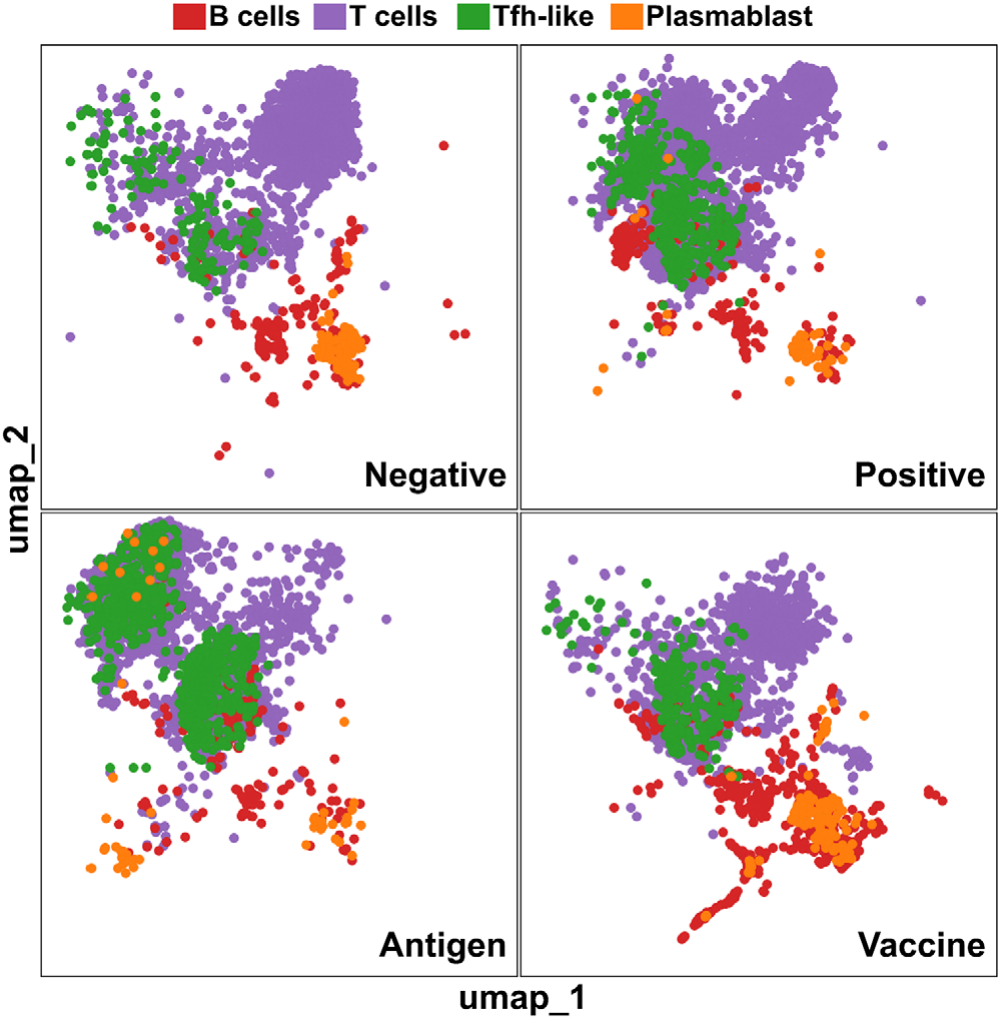
Stimulus‐dependent high‐dimensional UMAP visualization of immune cell populations. High‐dimensional flow cytometry data from all donors were concatenated and visualized using uniform manifold approximation and projection (UMAP). The immune subsets projected here: T cells (CD4+), B cells (CD19+), Tfh‐like cells (CD4+ CXCR5+ IL21R+ ICOS+ PD1+), and Plasmablasts (CD19+ CD20+ CD27+ CD38+). Each panel represents platform stimulation conditions as mentioned. In the negative (no stimuli) condition, a higher T cell population, with a few Tfh‐like cells, was observed, and plasmablasts were found, which could be the isolated population, and a lower B cell population was observed. The positive (5 µg/mL anti‐CD40 + 1 µg/mL IL‐4) condition resulted in a distinct remodeling of the immune landscape, characterized by an expanded T cell compartment with an increased proportion of Tfh‐like cells and a relative enrichment of B cells compared to the negative condition. Antigen (1 µg/mL SEB (staphylococcal enterotoxin B)) condition produced a landscape largely dominated by T cells, the majority of which displayed a Tfh‐like phenotype, and fewer B cells and plasmablasts. Vaccine (2.64 µg/mL Fluzone) condition generated a qualitatively distinct immune landscape, with proportionate representation of B and T cells and the highest relative abundance of plasmablasts.

### 2.6 High‐Dimensional UMAP Visualization of Immune Cell Populations in Response to Vaccine Stratified by Age and Donors

To understand and link donor‐specific vaccine‐induced IgG output to underlying cellular composition, we examined high‐dimensional UMAP projections of immune subsets on day 6 of culture (Figure 5). The immune subsets projected here are T cells (CD4+), B cells (CD19+), Tfh‐like cells (CD4+ CXCR5+ IL21R+ ICOS+ PD1+), and Plasmablasts (CD19+ CD20+ CD27+ CD38+) under vaccine (Fluzone) condition. Each panel represents age (aged; young) and donor identity (A1–A3; Y1–Y3) as mentioned. The collective/concatenated immune subset profile from young donors from the platform generated a relatively balanced representation of B and T cells, with a clear Tfh‐like population and detectable plasmablasts. In contrast, aged donors displayed a T‐cell‐dominant landscape with reduced B cells and fewer plasmablasts. These cellular differences align broadly with each donor's antibody response trajectory.

**FIGURE 5:**
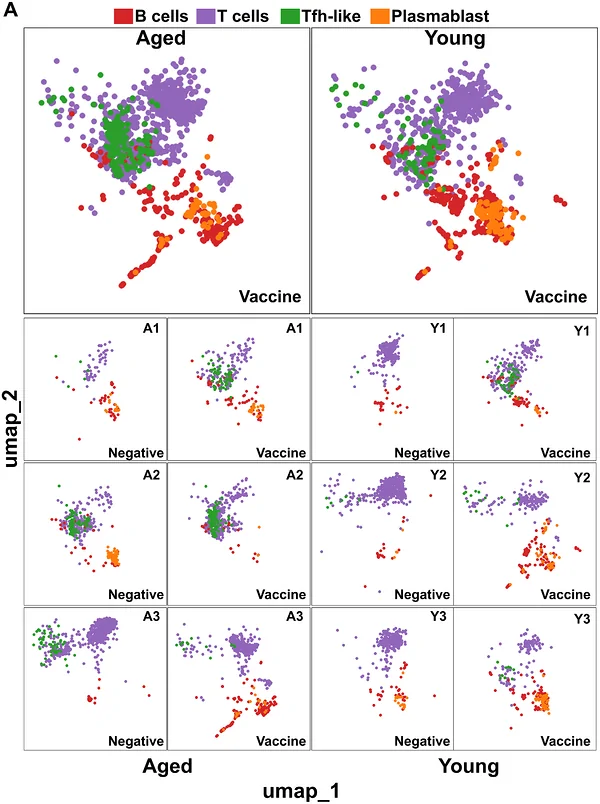
High‐dimensional UMAP visualization of immune cell populations in response to vaccine stratified by age and donors. High‐dimensional flow cytometry data from all aged and young donors were concatenated and visualized using uniform manifold approximation and projection (UMAP). The immune subsets projected here: T cells (CD4+), B cells (CD19+), Tfh‐like cells (CD4+ CXCR5+ IL21R+ ICOS+ PD1+), and Plasmablasts (CD19+ CD20+ CD27+ CD38+). Each panel represents platform age (aged; young) and donor identity (A1–A3; Y1–Y3) as mentioned. The collective/concatenated immune subset profile from young donors from the platform generated a relatively balanced representation of B and T cells, with a clear Tfh‐like population and detectable plasmablasts. In contrast, aged donors displayed a T‐cell‐dominant landscape with reduced B cells and fewer plasmablasts. These cellular differences align broadly with each donor's clinical serological trajectory, supporting a biological rather than artifactual observed variability. To better characterize the immune response, we compared vaccine‐treated organoids against a negative control. The presence of these populations in stimulated conditions versus their relative absence in unstimulated controls suggests an active, stimulus‐dependent remodeling of the immune landscape within the platform. Notably, Donor Y3 showed a robust increase in a plasmablast‐like population, demonstrating the transition from memory B cells to antibody‐secreting cells observed in clinical cohorts.

Aged donors displayed varying clinical antibody titers despite being classified as non‐responders, which is also observed in the immune landscape and in vitro IgG output. Donor A1 (Responder; T0 titer 80 and T2 titer 640, Figure 3B) exhibited B and T cells with a sizable proportion of Tfh‐like cells and a discernible plasmablast island. The platform IgG secretion peaked at day 4 and declined thereafter, consistent with a rapid but transient burst of plasmablast activity rather than sustained antibody secretion over the platform time frame. The observations suggest that, despite age‐related constraints, sufficient B cell targets and functional Tfh‐like help are present to support robust yet short‐lived plasmablast output. Donor A2 (Non‐responder; titer T0 titer 80 and T2 titer 80, Figure 3B) immune landscape is dominated by T cells, many of which map to a Tfh‐like phenotype, but with a striking scarcity of B cells and plasmablast population. This pattern suggests that, despite the presence of Tfh‐like phenotypes, insufficient B cell survival may represent a limiting factor for mounting an immune response. The observations are consistent with both poor in vivo seroconversion and negligible IgG secretion in the platform. Donor A3 (Non‐responder; titer T0 titer 160 and T2 titer 320, Figure 3B) displays a reasonable B:T ratio with some plasmablast differentiation but few Tfh‐like cells. The presence of B cells and a few plasmablasts suggests activation of B cells, but the scarcity of Tfh‐like cells may limit the magnitude of class‐switching and antibody secretion. Notably, the platform IgG secretion was negligible, which aligns with the cellular composition and a blunted but rising in vivo antibody titer. Overall, the results highlight the complexity of age‐associated immune responses, in which partial cellular engagement does not necessarily translate to robust functional output (Figure 5). Despite all young donors being classified as vaccine responders, substantial donor‐to‐donor variability is observed in both immune subset composition, functional output, and clinical antibody titers. Donor Y1 (Responder; T0 titer 40 and T2 titer 160, Figure 3B) shows a balanced B–T ratio with an immune composition enriched for Tfh‐like cells and relatively low plasmablasts. This pattern likely reflects a coordinated follicular‐associated interaction wherein sustained Tfh helps support antibody production. While canonical GC features such as somatic hypermutation were not assessed, the spatial and phenotypic alignment of Tfh‐like and B cell populations recapitulates key aspects of the follicular immune microenvironment. Alternatively, plasmablasts may have downregulated surface markers used for identification. Nonetheless, the observations are consistent with the progressive increase in antibodies as well as platform IgG secretion trajectories. Donor Y2 (Responder; titer T0 titer 160 and T2 titer 640, Figure 3B), despite a strong titer boost, exhibited a modest IgG accumulation in the platform supernatant. The immune landscape displays sizeable B cells with a large fraction differentiated to plasmablasts but relatively few Tfh‐like cells. Donor Y3 (Responder; T0 titer 40 and T2 titer 320, Figure 3B) demonstrated a B‐cell‐dominated composition with a high frequency of plasmablast‐like cells despite low IgG accumulation, suggesting commitment to the secretory lineage prior to maximal antibody production. This discordance likely reflects differences in the kinetics of plasmablast maturation and antibody secretion within the GCLO model rather than a failure of the platform itself. At day 6, the GC reaction is in its early phase, during which plasmablasts may have differentiated but not yet reached the peak antibody secretory capacity.

Overall, the strong in vivo response may reflect time differences, differences in per‐cell secretion capacity, or the antibody was produced by long‐lived plasma cells in bone marrow, which is not represented in PBMC culture (Figure 5). To distinguish between the survival of pre‐existing cells and active differentiation, we compared vaccine‐treated organoids against the negative condition (day 6). The selective expansion of plasmablast islands specifically under vaccine stimulation, particularly in Donor Y3, indicates a vaccine‐driven differentiation program rather than mere persistence of the baseline populations. The UMAP landscapes largely align with each donor's in vivo serological trajectories. Donors either expressed a response within the platform through accumulation of IgG in culture supernatent or higher expression of plasmablast phenotype (Figure 5). Notably, these analyses reveal that age‐related immune dysfunction is heterogeneous and donor‐specific, underscoring the value and fidelity of the platform in donor‐resolved immune profiling for understanding variability in vaccine response.

## 3 Discussion

The generation of high‐affinity antibody responses relies on precise cellular interactions within GCs, specialized microanatomical structures of secondary lymphoid organs [1, 3, 29]. While in vivo studies have identified many of the human molecular and cellular requirements of GC reactions, experimental systems that enable controlled interrogation of human GC‐like processes remain limited by architectural complexity, reliance on animal models, or the need for complex engineered scaffolds. Understanding the sources of inter‐individual variability in vaccine‐induced immune responses remains a central challenge in human immunology, particularly in the context of aging.

Among ECM components, fibronectin occupies a central role due to its abundance, multifunctionality, and dynamic regulation during immune activation. Fibronectin contains multiple binding domains for integrins, growth factors, cytokines, and other matrix proteins, enabling it to act as both a structural scaffold and a signaling hub [22, 23, 30]. Importantly, fibronectin fibrils are mechano‐responsive, i.e. cell generated forces stretch and unfold fibronectin modules, altering the exposure of binding sites and modulating interactions with immune cells and soluble mediators [20, 21]. These properties allow fibronectin to translate cellular forces into context‐dependent biochemical cues.

Consistent with this role, fibronectin is strongly upregulated and reorganized during inflammation and immune responses, where it serves as a permissive substrate for immune cell migration. In vivo imaging studies have shown that T cells migrate along fibronectin fibers in inflamed tissues via integrin‐dependent mechanisms, actively deforming the ECM to facilitate interstitial scanning. Disruption of fibronectin polymerization impairs T cell motility and alters immune cell accumulation, highlighting its functional importance in immune organization [30].

A central finding of the study is that a minimal ECM environment, without predefined spatial patterning or stromal cell engineering, is sufficient to enable self‐organization of moDCs, memory CD4+ T cells, naïve B cells into stable multicellular GCLOs that reproducibly exhibited features associated with early follicular reactions, including coordinated T–B localization, enrichment of CXCR5 expressing lymphocytes, and differentiation toward memory like and antibody‐secreting B cell states. Notably, GCLO formation and expansion occurred independently of specific stimulation, indicating that the ECM provides permissive biophysical cues that support immune cell interaction rather than instructing a particular activation state.

The type of stimulus identity strongly shaped the cellular composition and functional output of GCLOs. Positive condition favored B cell differentiation and antibody secretion, consistent with its known role in directly licensing B cell activation and class switching. SEB condition induced pronounced T cell enrichment and high frequencies of CXCR5+ T cells, yet resulted in comparatively limited B cell differentiation and IgG production. This dissociation highlights a key biological insight, follicular positioning cues and T cell activation alone are insufficient to drive efficient humoral output in the absence of appropriate B cell signals. In the vaccine condition, optimal T–B–APC interactions lead to the emergence of a more organized architecture characterized by a distinct B‐cell zone surrounded by T cells and APCs, consistent with a GC–like organization. This interaction consistently produced intermediate phenotypes, preserving both T–B balance and functional antibody secretion, thereby recapitulating features expected of physiologically relevant immune activation. Aged donors displayed strikingly heavy T cell landscapes with reduced B cell and fewer plasmablasts. The results align with the hallmarks of immunosenescence, such as diminished B cell reserve, impaired Tfh support, and constrained plasma cell differentiation. All young donors were clinical responders, but the cellular correlates of their vaccine‐induced IgG responses were heterogeneous, revealing multiple ‘successful’ immunological architectures. It is important to acknowledge that donor‐platform concordance was not absolute. Donor Y3, a clinical vaccine responder with an eight‐fold titer increase, showed minimal IgG secretion in GCLO culture at day 6. This discordance likely reflects a kinetic limitation of the current timepoint rather than an absence of immune activation. The high plasmablast‐like frequency in Y3 GCLOs suggests active differentiation that may not have been translated to measurable secretory output within the observation window. Future studies examining GCLO outputs at multiple timepoints beyond day 6 will be important to determine whether extending the culture period improves concordance with clinical responder classification.

The emergence of CXCR5+ T and B cells within the platform underscores the system's ability to support chemokine receptor programs. However, the observed variation in CXCR5 and CD27 expression across conditions further illustrates that phenotypic markers must be interpreted in context. For instance, high CD27 under unstimulated conditions likely reflects retention of pre‐existing memory B cells rather than active differentiation. Whereas reduced CD27 expression under antigen stimulation may indicate insufficient survival or maturation cues for sustained B cell output. These findings emphasize the value of integrating phenotypic, spatial, and functional readouts when evaluating follicular‐associated behavior in vitro.

Age‐dependent differences were preserved across multiple levels of analysis. Aged donors exhibited altered T–B cell balance, reduced frequencies of differentiated B cell subsets, and diminished vaccine‐induced IgG production, despite retaining or increasing CXCR5+ T cell frequencies. Notably, the platform mirrored the in vivo responsiveness of an aged donor, demonstrating its ability to preserve inter‐individual variability. This feature is thus relevant for studying heterogeneous human immune responses, where variability is biologically meaningful and not noise. The observed patterns are broadly consistent with established features of immunosenescence and impaired humoral immunity in aging; however, larger and more diverse donor cohorts, including intermediate age groups, will be required to validate these findings and define the cellular and molecular mechanisms underlying age‐associated differences in GCLO behavior.

The study has several important limitations. (i) The sample size is modest, particularly for age‐stratified analyses; thus, the findings are interpreted as descriptive rather than statistically definitive. The emphasis on donor‐wise kinetics and qualitative consistency across readouts was aligned with the exploratory nature of the platform. (ii) The system does not incorporate follicular dendritic cells, stromal chemokine gradients, or vascular inputs essential for sustaining a mature germinal center reaction in vivo. Thus, the conclusions are restricted to early follicular‐like organization and antibody production rather than affinity maturation or clonal selection. (iii) The phenotypic characterizations relied on flow cytometry and CSLM with no transcriptional signatures, limiting the resolution of underlying molecular states. It is important to note that GCLO‐derived IgG measurements represent local antibody secretion into organoid culture supernatant from a defined cell input, while clinical serum titers reflect the integrated systemic humoral immune response following in vivo vaccination. These are not equivalent measurements, and the observed correspondence between GCLO IgG trends and clinical titer classification should be interpreted as a preliminary correlative finding rather than a quantitative equivalence.

While acknowledging these limitations, this study establishes a robust and biologically informative platform for interrogating coordinated T–B cell interactions and functional antibody production in a controlled ex vivo setting. Importantly, the system is not intended to recapitulate a fully mature GC, which in vivo depends on specialized stromal and vascular networks, follicular dendritic cells, and spatially organized antigen deposition [31, 32]. Rather, it captures a defined subset of organizational and signaling features that are sufficient to support lymphocyte self‐organization, productive T–B interactions, and IgG secretion in a reproducible and scalable format. Despite its simplified architecture, the platform reliably preserves age‐associated differences in cellular organization, B‐cell differentiation, and antibody output, demonstrating its sensitivity to biologically meaningful variation in immune function. The ability to observe coordinated, time‐dependent immune behaviors rather than isolated cellular readouts represents a key strength and distinguishes this approach from conventional co‐culture assays. As such, this model provides a valuable intermediate system that bridges standard in vitro cultures and complex in vivo studies, enabling mechanistic hypothesis testing, comparative immune profiling, and iterative refinement. With future integration of stromal components, naïve‐B cells, and transcriptional profiling, this platform has strong potential to evolve into a versatile tool for studying immune dysfunction and therapeutic modulation across aging and disease contexts.

## 4 Methods

### 4.1 Cellular Isolation and Seeding

Deidentified cryopreserved PBMC samples were obtained from both young (≤ 40 years; *n* = 3) and older (≥ 60 years; *n* = 3) individuals from an ongoing University of Miami Institutional Review Board approved study (IRB protocol # 20200752). Each participant received the Fluzone Quadrivalent Influenza Vaccine corresponding to that year. PBMC collected at day 28 post‐vaccination were used for this study. On Day ‐8, one PBMC vial is used to isolate monocytes using the EasySep Human Monocyte Isolation Kit (StemCell Technologies, #19359), which negatively selects CD14^+^ monocytes. Isolated monocytes are then cultured in ImmunoCult Dendritic Cell Medium (StemCell Technologies, #19359) supplemented with ImmunoCult Dendritic Cell Differentiation Supplement (StemCell Technologies, #10988) for differentiation to dendritic cells. On Day ‐5, the medium was replaced by centrifugation at 300 g for 10min. On Day ‐3, ImmunoCult Dendritic Cell Maturation Supplement (StemCell Technologies, #10989) to promote maturation of the differentiated dendritic cells. On Day ‐1 of the co‐culture experiment, two additional vials of PBMC from the same donor are thawed and cultured for 2–3 h in complete RPMI‐1640 medium at 37°C with 5% CO_2_. The B cells were negatively selected and isolated using the EasySep Human B cell Isolation Kit (StemCell Technologies, #17954) from one flask. The memory CD4^+^T cells were negatively selected and isolated using the EasySep Human Memory CD4^+^T cell Enrichment Kit (StemCell Technologies, #19157) from the other flask. All cell types were counted using a hemocytometer prior to seeding. Mature DCs, CD4^+^ T cells, and B cells were combined in a 1:1:1 ratio (50 000 cells each) and suspended in 2 mg/mL fibronectin to get a final concentration of 1 mg/mL. The cells suspended in fibronectin are seeded onto a flat bottom, uncoated 96‐well plate (Corning #3881) and allowed to adhere for 1–2 h at 37°C with 5% CO_2_ and 5% humidity. Subsequently, a complete RPMI‐1640 (RPMI+ 10% FBS (Gibco #A5209501) + 1% PenStrep (Gibco #15140122)) culture medium was added, and plates were returned to the incubator for overnight culture. On Day 0, specific stimuli i) No Stimuli: Negative Condition (ii) 5 µg/mL anti‐CD40 + 1 µg/mL IL‐4: Positive Condition, (iii) 1 µg/mL SEB (Staphylococcal Enterotoxin: Antigen Condition, (iv) 2 µg/mL Fluzone: Vaccine Condition was added to the respective wells (Figure 1B). We ensured that the Fluzone lot corresponded to the same year as the donor's vaccination. For each donor, three replicate wells were established per stimulation condition. One well per condition was designated for live‐cell imaging and confocal microscopy; two wells per condition was used for flow cytometry following organoid dissociation. The supernatant from the flow cytometry wells were used for IgG analysis.

### 4.2 Cellular Staining for Confocal Microscopy

The co‐cultures in various conditions were fixed with 4% PFA (paraformaldehyde) (Electron Microscopy Sciences #15710) and permeabilized using 0.1%w/v of Saponin (Sigma–Aldrich #80058‐630) + 0.01% Triton X at RT/ 5 min. Saponin permeabilization keeps membrane‐associated proteins in place, while allowing antibodies into the cell where they can bind to intracellular epitopes. The cultures were washed with PBS at RT/ 5 min and blocked using 2% BSA (bovine serum albumin in PBS) (Sigma–Aldrich #A2153‐50G) at RT/1 h. The sections were rinsed with 1X PBS (Gibco #10010023) and stained by incubating with fluorophore‐labelled antibodies directed against CD27‐Cy7 (BD Bioscience, 563786), CXCR5‐AlexaFluor 647 (BD bioscience, 5588113), CD16‐FITC (Biolegend, 300405) (Figure S2), CD19‐PC5.5 (Beckman Coulter, A66328), and CD3‐BV510 (BD Bioscience, 563109) at RT/1 h (Figure 2A.ii and Figure S1). The images were acquired using a Carl Zeiss laser scanning confocal microscope (LSM780) and exported as .czi files. The files were processed in FIJI (ImageJ). To correct the systematic error of contrast loss caused by the microscope objective lens, we performed deconvolution on the images captured [33]. We used the metadata to generate a z‐stack of theoretical point spread function (PSF) and used that as a reference for deconvolution using the Deconvolution Lab2 plugin in ImageJ. We used the Richardson‐Lucy algorithm, and 10 iterations were carried out. The deconvolution was performed for each channel in the z‐stack, then merged, and contrast and brightness were adjusted.

### 4.3 Immunoglobin G (IgG) Quantification

The culture supernatants (no dilutions performed) collected from the platform culture are assessed for total IgG concentration using the Human IgG ELISA kit (Bethyl Laboratories, Inc, E88‐104_230411). The assay was performed according to the manufacturer's instructions, ELISA was conducted in a 96‐well plate format, and absorbance was measured at 450 nm using a microplate reader (Beckman Coulter). To quantify IgG concentrations, a standard curve was generated using known concentrations provided in the kit. Spectrophotometric absorbance values were analyzed using R (version 4.4.1, R Foundation for Statistical Computing, Vienna, Austria). A custom script was used to interpolate sample concentrations from the standard curve using the four‐parameter logistic (4PL) model. Heatmaps for IgG concentration (ng/mL) across all conditions was mapped out. LOESS regression was applied to stratify the IgG kinetics by age. Given the exploratory sample size (*n* = 3 per group), age‐stratified analyses were interpreted descriptively, and no inferential statistical testing was performed.

### 4.4 Phase Contrast Microscopy and Analysis

Phase‐contrast images of live cells were acquired using the EVOS XL core imaging system (Thermo Fisher Scientific) equipped for transmitted light and phase‐contrast microscopy. Samples were imaged directly in their culture well system with 4x objective and the corresponding 4/10PH condenser annulus. The imaging was performed under consistent illumination settings across all conditions. GCLOs were focused using the live preview and fine focus controls, the frame was frozen, and images were acquired using the integrated high‐sensitivity digital camera and system software at native camera resolution. For each condition, 2 fields were acquired to capture the GCLOs present in the entire well. The acquired images were exported as TIFF at 3MP resolution and processed in FIJI (ImageJ). The individual fields for each condition were stitched using pairwise stitching in Fiji, and the linear blending method was used to fuse the images. The brightness and contrast were adjusted linearly across the stitched file to enhance resolution. No non‐linear transformations or local contrast enhancements were applied. All images were adjusted using a custom macros script to ensure uniformity. Within a given experiment, identical post‐processing parameters were applied to all images from the same acquisition batch.

### 4.5 Flow Cytometry

The cells (on day 6) from each condition of the platform were harvested by gentle pipetting to dislodge and subsequently passed through a 20 µm cell strainer to remove the fibronectin matrix and break up the GCLOs. The filtered cellular suspension was washed and stained with LIVE/DEAD Blue, followed by surface staining was performed using a predefined panel of fluorochrome‐conjugated antibodies. Cells were stained according to standard protocols in staining buffer and acquired fresh without fixation. The stained samples from each condition were analyzed on a Cytek Aurora spectral flow cytometer on day 6 of the experiment. Single‐stained controls (beads) were acquired on the cytometer for each fluorochrome, along with fluorescence minus one (FMO) controls for key markers to aid in gating. The acquisition of samples from various was performed across independent acquisition days using identical instrument settings, and a common reference control and calibration beads were used. We exported unmixed and uncompensated data as .fcs files for downstream analysis. The files were analyzed in OMIQ (vers), and manual compensation was applied using single‐stained controls. All samples were transformed using an arcsinh function with a cofactor of 6000, applied uniformly across the dataset. Manual quality control gating was applied to exclude debris, doublets, and dead cells, followed by gating the relevant lineage as shown in Figure S3. No downsampling was performed. For each gated population, frequencies were calculated as a percentage of total live cells and as a percentage of the respective parent gate (Figure 2B,C). Since the sample size is *n* = 6, no formal statistical tests were applied, and results are presented as descriptive.

High‐dimensional Uniform Manifold Approximation and Projection (UMAP) in OMIQ was performed on all compensated and transformed files. UMAP was computed with the following parameters: number of neighbors = 15, minimum distance = 0.4, number of components = 2, Euclidean metric, learning rate = 1200 epochs, random seed = 6788, and spectral internalization. No clustering algorithms were applied. UMAPs were generated for all samples or selected donors with predefined feature subsets, depending on the analysis, as mentioned in the figure legends. All analyses were performed using identical preprocessing and visualization settings to ensure comparability across experiments.

### 4.6 Live Cell Imaging of Fluorescently Labeled Immune‐Cell Subsets

Live‐cell imaging was performed using Incucyte SX5 (Sartorius) live‐cell‐analysis system within a standard tissue‐culture incubator (37°C/5%CO_2_/ 5% humidity). Incucyte Cytolight Rapid Green Dye (Sartorius #4705) was used to label T cells, and Incucyte Cytolight Rapid Orange Dye (Sartorius #4839) was used to label B cells. The protocol for staining was performed according to the manufacturer's instructions, and the dye concentration optimization was conducted as a separate experiment prior to the final experiments. Each well was divided into eight fields of view, and images acquired were auto‐focused at 10X every 2 h from days 0 to 6. The imaging was conducted using phase contrast, green fluorescence, and orange fluorescence channels with identical acquisition settings across samples and conditions. Time lapse image series were exported as TIFF files and processed in FIJI (ImageJ). A custom script was written to ensure uniform and auto‐processing of all files into video format. Briefly, we enhanced contrast, merged channels, and converted the stacks to avi file. Since moDCs were only visible in phase contrast, we pseudo stained them as blue for visualization purposes.

## Author Contributions

**Giancarlo Asencio**: investigation. **Ashutosh Agarwal**: data curation, conceptualization, investigation, funding acquisition, methodology, validation, visualization, writing – review and editing, software, formal analysis, project administration, supervision, resources. **Bhumi Suthar**: investigation, writing – original draft, methodology, validation, visualization, writing – review and editing, software, formal analysis, data curation. **Carlos Gomez**: investigation. **Suresh Pallikkuth**: conceptualization, investigation, funding acquisition, methodology, validation, visualization, writing – review and editing, software, formal analysis, project administration, resources, supervision, data curation.

## Conflicts of Interest

The authors declare no conflicts of interest.

## Supporting information




**Supporting File**: advs76335‐sup‐0001‐SuppMat.docx.

## Data Availability

The data that support the findings of this study are available on request from the corresponding author. The data are not publicly available due to privacy or ethical restrictions.
