# Supplementary material for: Engineering Human Donor Derived Germinal Center‐Like Organoids (GCLOs) for Studying Immune Response to Vaccination
Source: Adv Sci (Weinh). 2026 Jul 3:e76335. Online ahead of print. doi: 10.1002/advs.76335 (PMC13334605; doi:10.1002/advs.76335)
Supplement: Supplementary file 1 — Supporting File: advs76335‐sup‐0001‐SuppMat.docx. [file ADVS-9999-e76335-s001.docx]

**Supplementary Information**

**Engineering Human Donor Derived Germinal Center-like Organoids (GCLOs) for studying Immune Response to Vaccination**

Bhumi Suthar^1^, Carlos Gomez^2^, Giancarlo Asencio^1^,
Suresh Pallikkuth^2^*, Ashutosh Agarwal^1,3^*

^1^ Department of Biomedical Engineering, University of Miami

^2^ Microbiology and Immunology, University of Miami Miller School of Medicine

^3^ Desai Sethi Urology Institute, University of Miami Miller School of Medicine

**Corresponding Authors:**

Suresh Pallikkuth, PhD

TEL: +1 305 243-5315, Email: SPallikkuth@miami.edu

Ashutosh Agarwal, Ph.D.

TEL: +1 305 243-8925, E-mail: A.Agarwal2@miami.edu


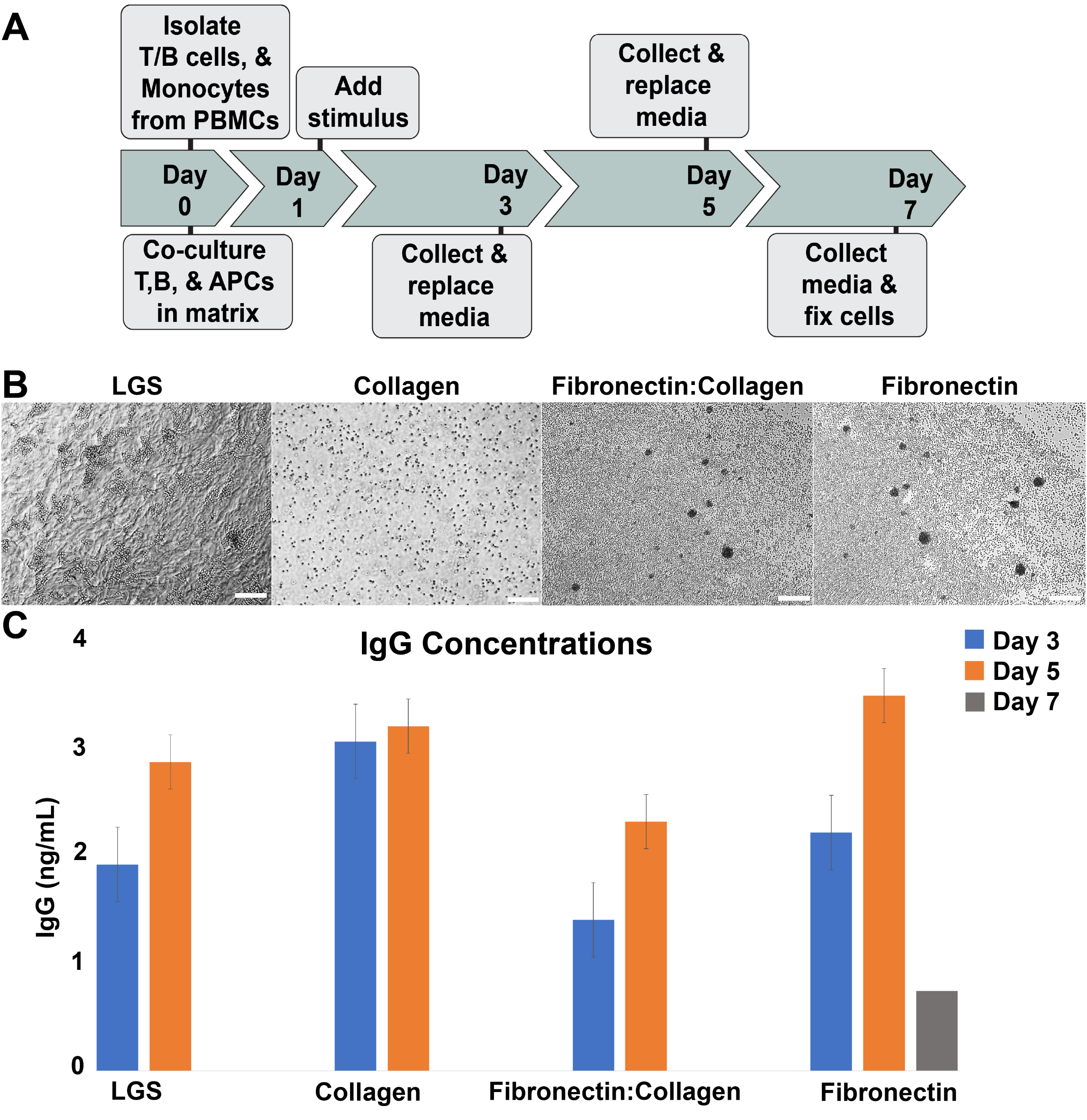


**Supplementary Figure 1: Optimization of ideal microenvironment for formation of GCLOs**

**(A)**Experimental workflow outlines the major steps in a timely manner. Immune cells such as Monocytes (CD11c+/CD14+), T cells (CD3+), & B cells (CD19+) were positively isolated from human donor-derived PBMCs (peripheral blood mononuclear cells) with flow cytometry. Monocytes played the role of APCs (antigen presenting cells). Isolated immune cells were co-cultured on a 48-well plate in respective microenvironments.

**(B)** Phase Contrast Microscopy images of GCLOs formed in various microenvironments. T cells, B cells, and APCs were co-cultured in respective microenvironments and stimulated with 5μg/mL anti-CD40 + 1μg/mL IL-4. We tested **Lyophilized Gelatin Scaffold (LGS)**: which was 5mm in diameter and 200μm thickness and ~50µm pore size, **Collagen solution**- 3.7mg/mL of rat tail collagen IV was used, **Fibronectin: collagen solution:** 1:1 ratio, **Fibronectin solution:** 1mg/mL of bovine derived fibronectin was used. Apart from collagen matrix, clusters were observed in all microenvironments. **(C)** IgG concentration accumulated in culture supernatant of various microenvironments was quantified using ELISA. Although each matrix produced detectable IgG, a sustained IgG concentration was observed in immune cells suspended in fibronectin.


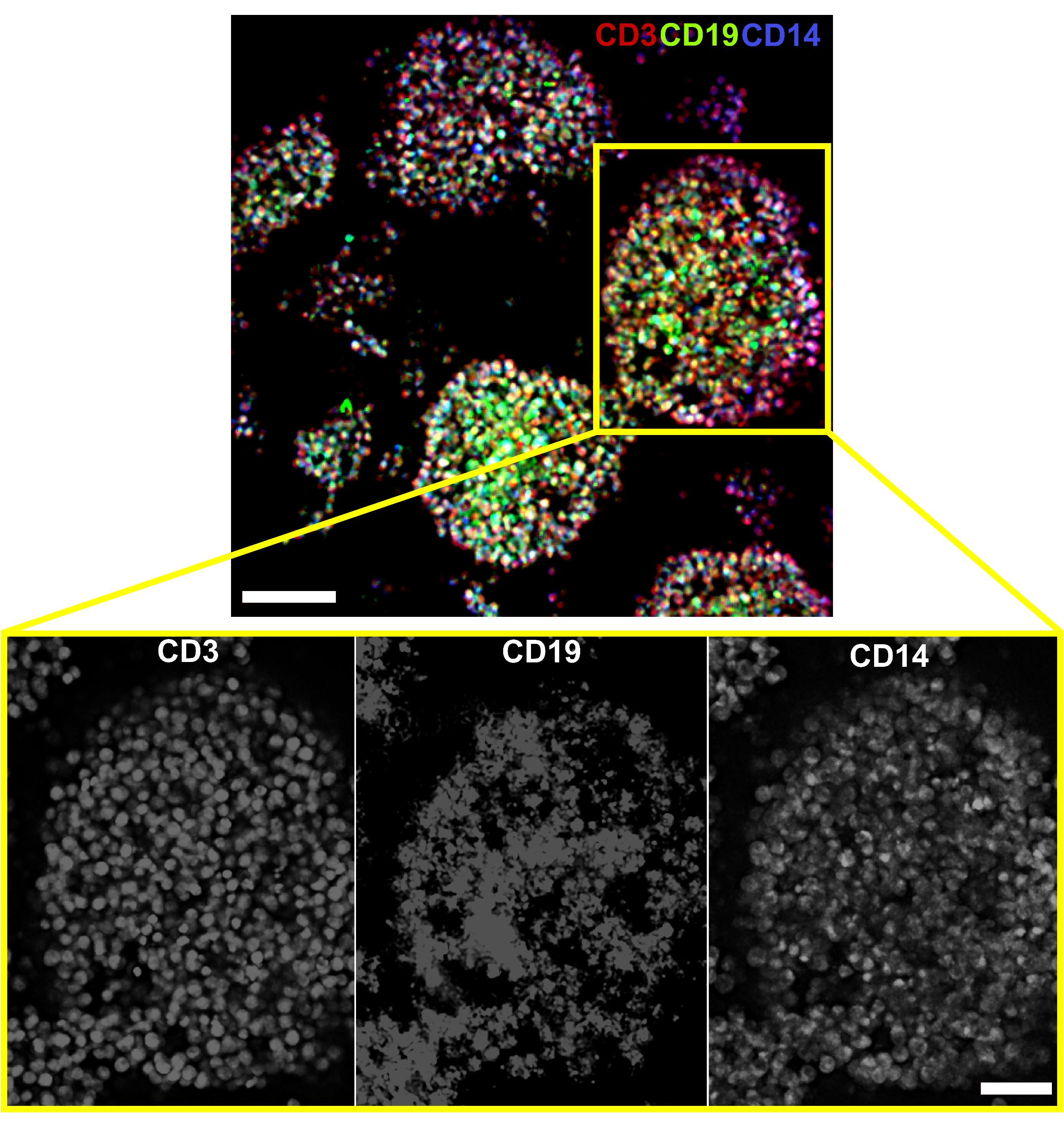


**Supplementary Figure 2: Confocal Microscopy of GCLOs.**

Multiple GCLOs with T cells (CD3 red), B cells (CD19 green), moDCs (CD14 blue) observed communicating with each other, scale bar represents 100μm. The binary grayscale representations of the highlighted GCLO illustrate the degree of intermixing and spatial overlap between immune subsets, suggesting active communication and coordination within the platform, scale bar represents 50μm.


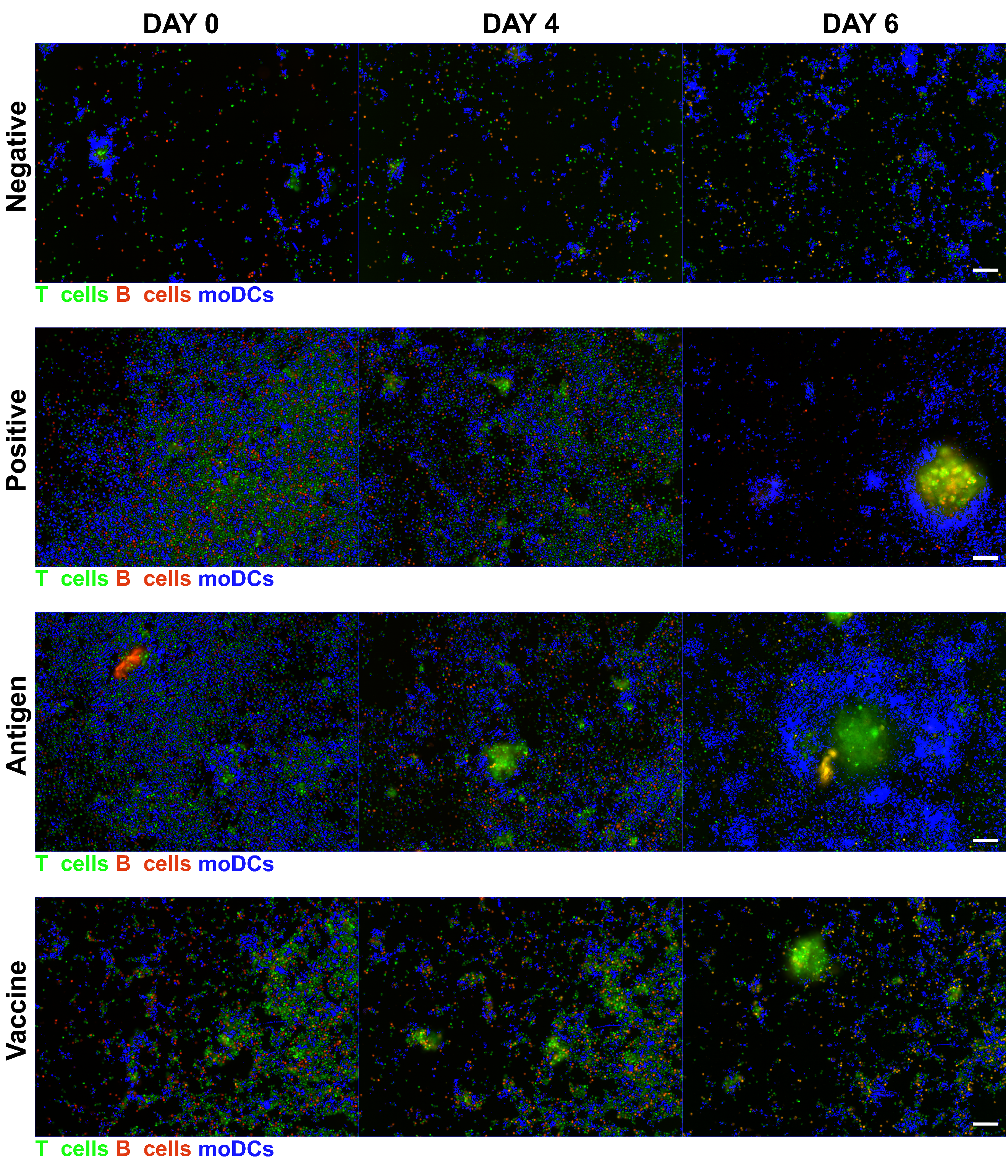


**Supplementary Figure 3**: **Live cell imaging of GCLOs across stimulation (Day 0 - 6)** To observe the interaction of T cells (green), B cells (red), and moDCs (blue) to GCLO formation we took images every 2 hours from Day 0 to Day 6 of the platform. Scale bar represents 100μm.





**Supplementary Figure 4: Platform supports differentiation of immune cells to GC-like effector phenotypes**

**(A)** Quantification of CXCR5+ T cells, CXCR5+ B cells, and CD27+ B cells across negative, positive, antigen, and vaccine conditions. **(B)** Quantification of age stratified frequencies under vaccine stimulation. **(C)** Representative immunofluorescence image illustrating spatial co-localization of CXCR5 (red), CD27 (green), & CD16 (blue) expressing cells within the cluster.


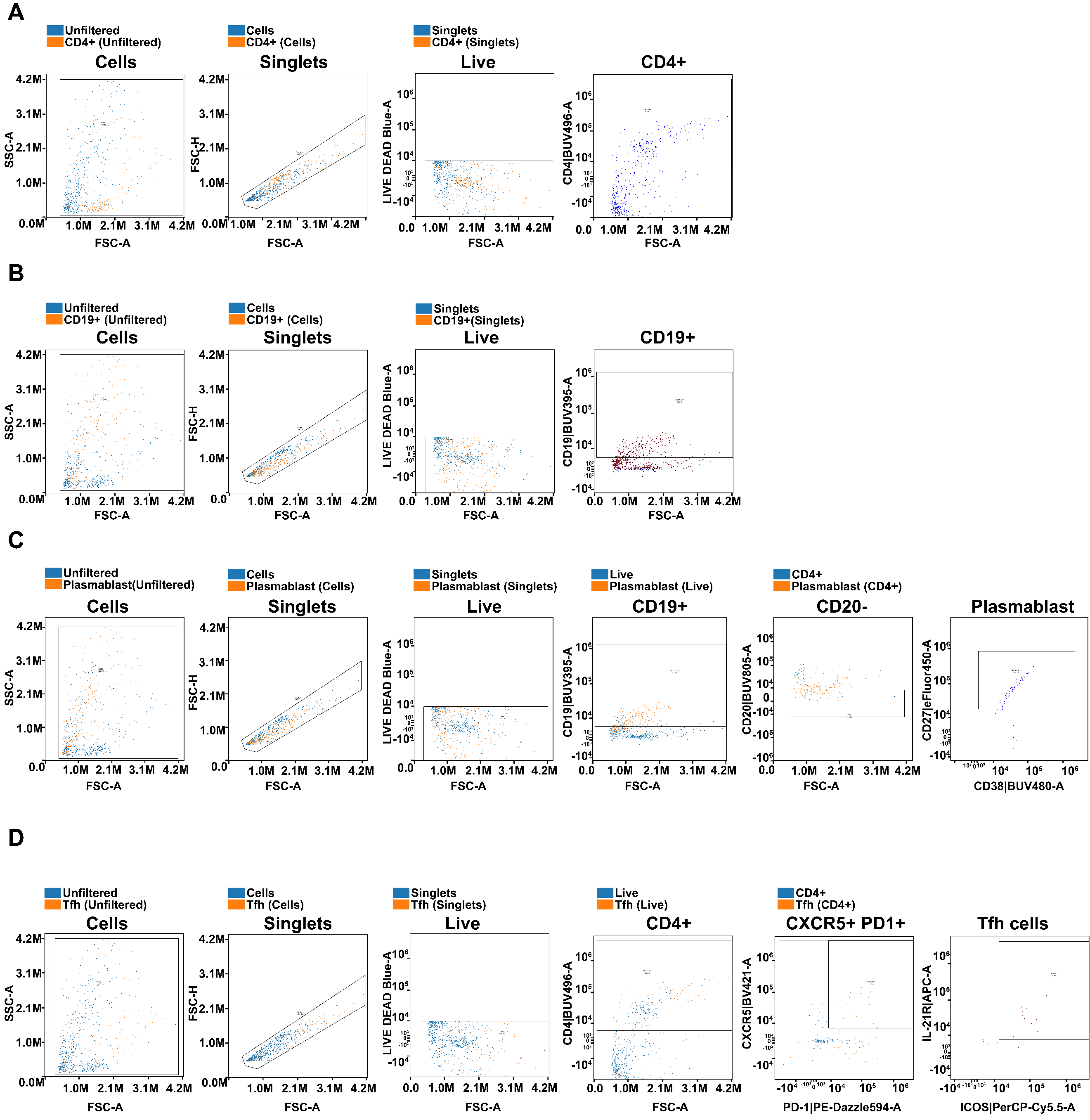


**Supplementary Figure 5: Gating strategy applied for various immune cell subsets performed in OMIQ**
